# Lemierre’s Syndrome: A Cloaked Dagger

**DOI:** 10.7759/cureus.36087

**Published:** 2023-03-13

**Authors:** Samuelson E Osifo, Akhabue G Umolu, Wayomi R Perera, David C Howlett

**Affiliations:** 1 Surgery, University Hospitals Sussex NHS Foundation Trust, Brighton, GBR; 2 Otolaryngology-Head and Neck Surgery, East Sussex Healthcare NHS Trust, Eastbourne, GBR; 3 Respiratory Medicine, University Hospitals Sussex NHS Foundation Trust, Brighton, GBR; 4 Interventional Radiology, East Sussex Healthcare NHS Trust, Eastbourne, GBR

**Keywords:** internal jugular vein thrombophlebitis, septic thrombophlebitis, fusobacterium necrophorum, tonsilitis, lemierre's syndrome

## Abstract

Lemierre’s syndrome is a condition characterized by septicemia, with bacteremia, thrombophlebitis of the internal jugular vein (IJV), and septic embolization to distant organs following a recent upper respiratory infection (URI). *Fusobacterium necrophorum, *an anaerobic Gram-negative rod, has been mostly implicated as the causative organism of this condition that tends to affect healthy teenagers and young adults. While once regarded as a disease of old, it has seen a resurgence in recent times, possibly due to antibiotic stewardship and current trends of reduced antibiotic use for URIs. It is important that the modern physician has a high index of suspicion, as well as the characteristic presentation of this potentially fatal illness. Current treatment guidelines are centered on the use of appropriate antibiotics, drainage of purulent collections when possible, and, in some situations, anticoagulants have been utilized. This study describes a case of a young lady who presented with symptoms of chest pain and deteriorating oxygen saturations following recent treatment for acute tonsillitis.

## Introduction

Lemierre's syndrome is an anaerobic postanginal septicemia - usually due to *Fusobacterium necrophorum* - causing thrombosis of the superior internal jugular vein (IJV) with distant septic emboli [1-4]. It can result in severe morbidity and mortality, sometimes despite appropriate antibiotic therapy, hence the need for a high index of suspicion and prompt treatment [5,6].

This study describes a patient who presented with pleuritic chest pain and deteriorating oxygen saturations following treatment for tonsillitis. Computed tomography (CT) scans were negative for pulmonary emboli but showed multiple septic emboli in the lung parenchyma and thrombi within the left IJV. Her condition resolved following an appropriate course of antibiotics and anticoagulants.

## Case presentation

A 25-year-old lady presented with a four-day history of sore throat, odynophagia, fever, and pain at the angle of both jaws. The patient was otherwise fit and well, with no significant past medical history of note, besides a documented penicillin allergy. She smoked five to 10 cigarettes per day. On examination, she had bilateral swelling of the tonsils with visible exudates and bilateral tender cervical lymph nodes, worse on the left. She was febrile with a temperature of 38.6°C and tachycardic with a heart rate of 100 beats per minute. Blood tests showed raised c-reactive protein, but other blood parameters were unremarkable. She was diagnosed with acute tonsillitis and treatment commenced with IV clarithromycin, non-steroidal anti-inflammatory drugs (NSAIDs), with benzydamine hydrochloride gargles, and a stat dose of dexamethasone. She improved clinically and was discharged home 24 hours later, on oral clarithromycin, NSAIDs, and benzydamine hydrochloride gargles.

Four days later, the patient returned feeling generally unwell and complained of bilateral pleuritic chest pain, with fever and dyspnoea. She was pyrexic (38.9°C), tachycardic, and tachypneic, with an obvious swelling of her left tonsil but no visible exudates. She had tender cervical nodes on the left side, and cardiopulmonary examination was positive for decreased air entry at the lung bases. Blood tests showed a marked leucocytosis with elevated c-reactive protein, alkaline phosphatase, D-dimer, and hypoalbuminemia.

**Figure 1:**
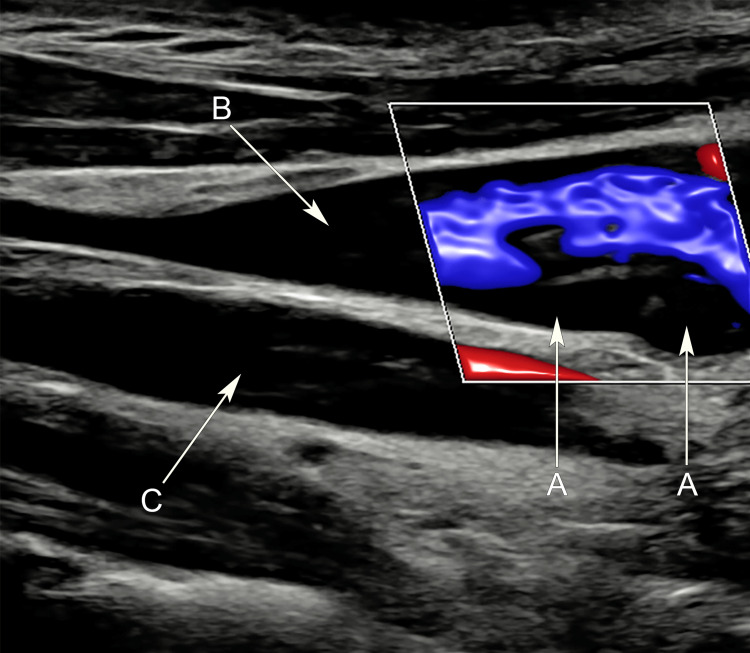
Longitudinal sonogram of the left neck Sonogram demonstrates a hypoechoic, non-occlusive thrombus within a dilated left internal jugular vein (arrow A). Patent internal jugular vein is shown cranial to the thrombus (arrow B), and common carotid artery (arrow C).

Burger investigation with a color Doppler ultrasound scan (USS) of the neck confirmed a non-occluding thrombus in the superior left IJV (Figure 1).

**Figure 2:**
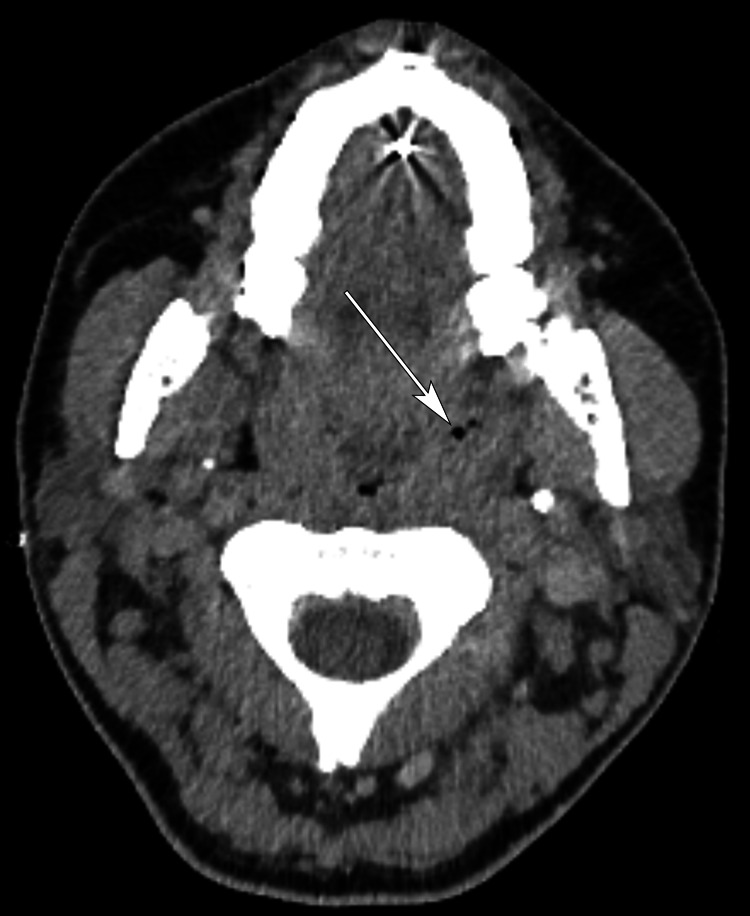
Postcontrast CT scan of the neck at the tongue base/tonsillar level There is symmetrical palatine tonsillar enlargement. No focal fluid collection is present, but bubbles of gas can be seen within the left palatine tonsil (arrow) consistent with infection with a gas-forming organism.

**Figure 3:**
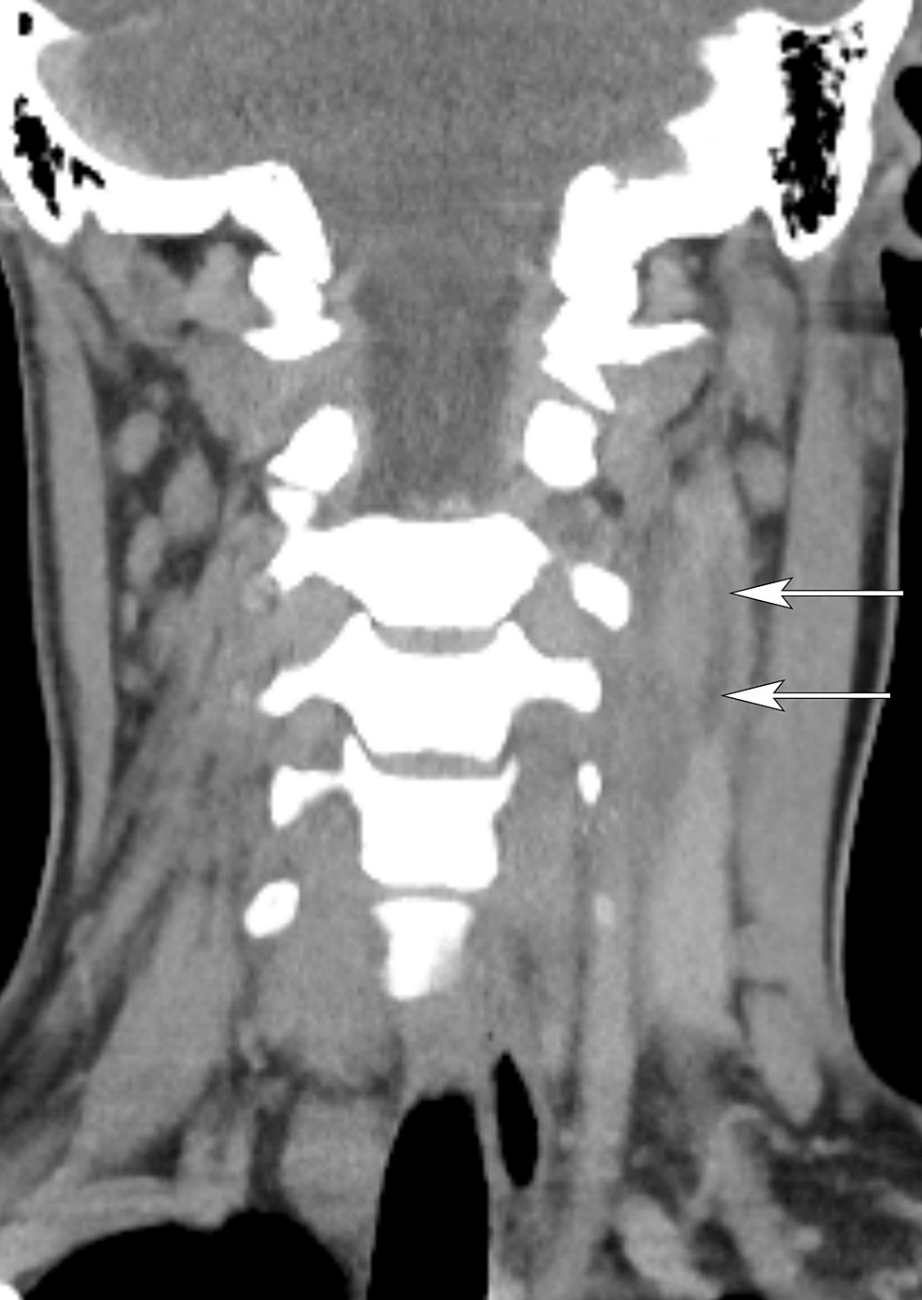
Coronal CT of the neck Coronal postcontrast CT through the left internal jugular vein confirms linear low-density thrombus (arrows).

**Figure 4:**
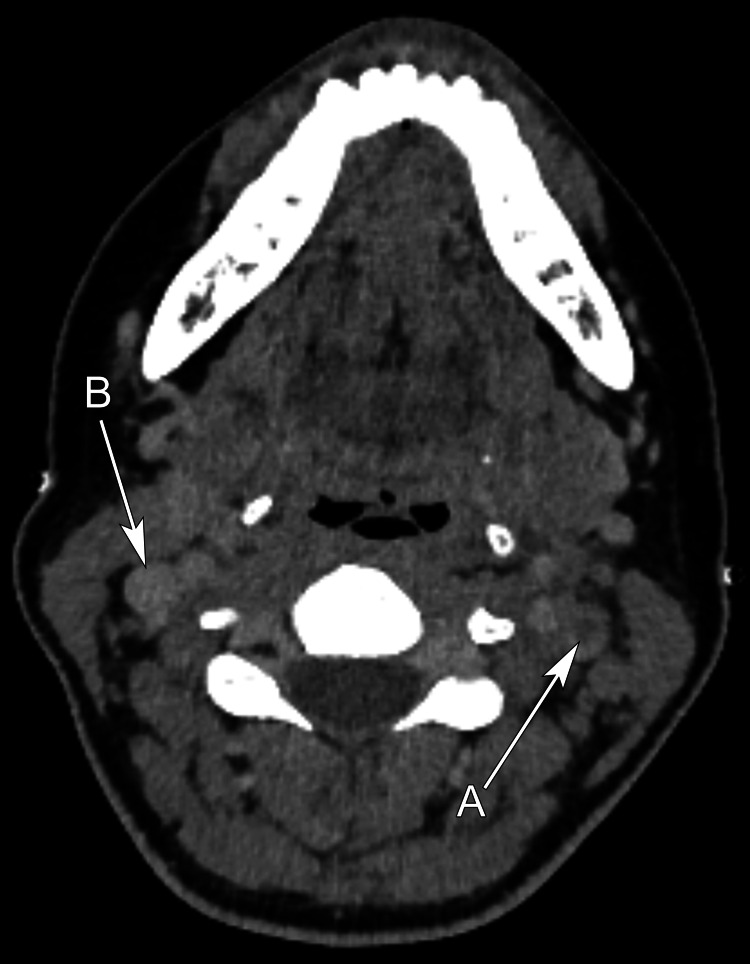
Axial CT postcontrast at the level of the upper neck This confirms a rounded focus of low attenuation in the left internal jugular vein (arrow A) in keeping with thrombus. Note normal enhancing right internal jugular vein (B).

**Figure 5:**
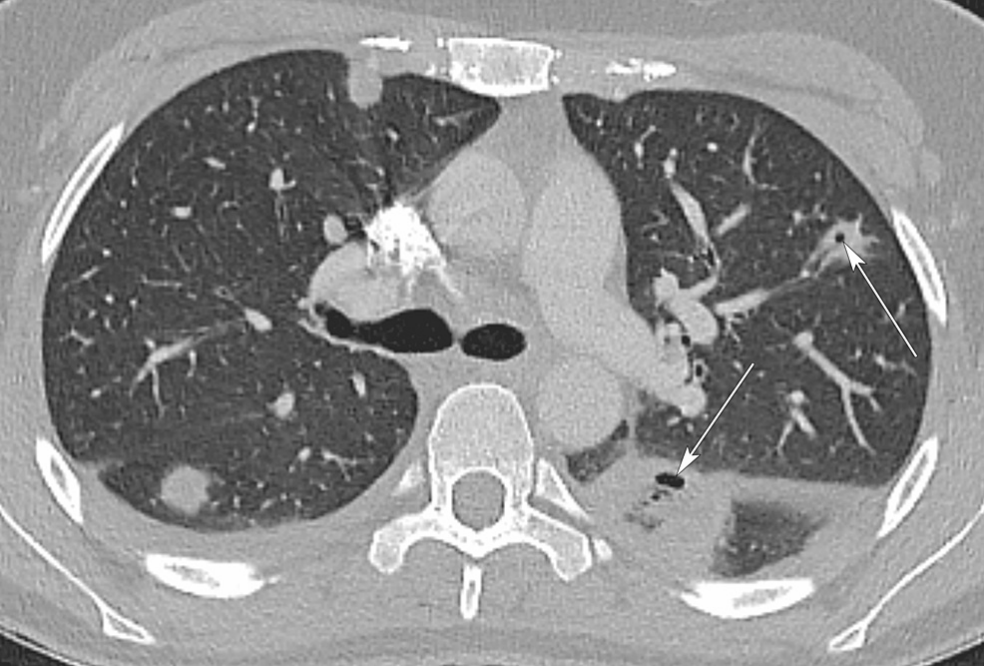
Axial CT pulmonary angiogram Axial CT image through the lungs at the level of the carina, lung window setting, showing multiple ill-defined soft tissue nodules in both lungs and sub-pleura. Multiple thick-walled lung cavitations can be seen (arrows), consistent with septic emboli.

Chest radiograph showed consolidation at both lung bases with a small left basal pleural effusion. CT neck confirmed mild asymmetric enlargement of the left tonsil, which contained bubbles of air suggestive of an infection (Figure 2), and a low-density thrombus within the superior aspect of the left IJV (Figures 3, 4). CT pulmonary angiogram (CTPA) was negative for pulmonary emboli but demonstrated multiple thick-walled lung cavitations, consistent with septic emboli (Figure 5). Blood cultures yielded growth of *Fusobacterium necrophorum* after 48 hours of incubation, but echocardiogram did not reveal any vegetations.

The patient was treated with IV ceftriaxone, metronidazole, analgesia and IV fluids, and anticoagulated with rivaroxaban, improving significantly, and was duly discharged home after 14 days to continue oral antibiotics for a further four weeks, and anticoagulation for a further 10 weeks. A follow-up CT chest six weeks later showed resolution of lung abnormalities. Repeat color Doppler USS showed a patent left IJV. She remains well and is under review although tonsillectomy is not currently under consideration.

## Discussion

Septic thrombophlebitis is characterized by venous thrombosis, inflammation, and bacteremia or fungemia [1] often with varying severity and possible clinical courses. While a number of distinct clinical conditions have been identified affecting several veins - peripheral veins, pelvic veins, portal veins, superior vena cava or inferior vena cava, internal jugular vein or dural sinuses - they all share one basic pathophysiology [4]. Lemierre’s syndrome is a rare and serious septic thrombophlebitis that could ultimately become fatal without prompt and effective treatment [5]. It is characterized by an acute primary oropharyngeal infection, suppurative thrombophlebitis of the IJV, septic embolization, and oftentimes a documented causal relationship with *Fusobacterium necrophorum*. This condition typically occurs in previously healthy teenagers and young adults with the majority affected (89%) aged between 10 and 35 years [6]. It has a male preponderance with a male-to-female ratio of 2:1 described in some studies [7].

While Lemierre’s syndrome is thought by some to be an uncommon illness, there is evidence that this condition has seen a resurgence of late [8]. This rise in incidence is likely a reflection of some key changes in medical practice; firstly, restriction in the use of antibiotics to treat upper respiratory tract infections and overconsumption of non-steroidal anti-inflammatory drugs during ear, nose, and throat (ENT) infections [8], and secondly, a tightening of the criteria for tonsillectomy. Risk factors for Lemierre’s syndrome include a previous history of recurrent pharyngitis/tonsillitis (as in our patient), sinusitis, dental procedures, and, in some cases, immunosuppressive comorbidities like diabetes mellitus and head and neck malignancies. The time from the beginning of the oropharyngeal infection and the onset of septicemia is approximately one week [9]. 

*Fusobacterium necrophorum*, an anaerobic Gram-negative, non-spore-forming bacillus is most commonly implicated. In a published case series, blood cultures of 68% of patients grew *Fusobacterium necrophorum*, 86% of cultures from patients with Lemierre’s syndrome grew *Fusobacterium necrophorum* or another Fusobacterium sp., and in all 90% grew anaerobic bacterium of some type [6]. Infection of the tonsil and the lateral pharyngeal space is typically polymicrobial, with thrombophlebitis in the draining tonsillar veins being the critical event [10]. While the pathogenetic mechanisms of *Fusobacterium necrophorum *infection are complex and not well defined, several toxins - including hemolysins, haemagglutinins, leukotoxins, endotoxins, and adhesins - have been implicated as virulence factors. Hemagglutinin, one of the toxins produced, has the ability to stimulate clot formation (and the organism multiplying within the clot) with subsequent embolic spread. Septic pulmonary emboli are almost always present and often lead to grave complications, including empyema, lung cavitation, and hypoxemia. Seldomly, septic emboli may transverse a patent foramen ovale and cause distant metastatic infections such as liver abscesses, osteomyelitis, and septic arthritis.

In patients presenting with fever, chills, rigors, in rare occasions, cranial nerve palsies, and a history of a recent oropharyngeal infection, with or without symptoms suggestive of embolic lesions or respiratory symptoms (including pleuritic chest pain, dyspnoea, hemoptysis), Lemierre’s syndrome should be suspected. Imaging investigations in addition to blood investigations should be promptly undertaken. A color Doppler USS of the neck is an accurate means of diagnosing IJV patency. Ultrasound evaluates the vein above the clavicle and is not the optimum technique for assessment of acute tonsillar infection and the deeper neck spaces, for which contrast-enhanced CT is recommended. A CTPA will show septic emboli in the lung parenchyma and help rule out a pulmonary embolism. 

A multidisciplinary approach is key in the treatment of patients with Lemierre’s syndrome. Invaluable contribution from microbiologists, infectious disease experts, hematologists, radiologists, and otolaryngologists is important to reach a prompt diagnosis and achieve therapeutic success [11]. The mainstay of management for Lemierre’s syndrome is early recognition and then prompt administration of IV antibiotics and surgical drainage of collections as appropriate. Prolonged therapy for three to six weeks is recommended to allow time for antibiotics (initially IV then subsequently oral) to penetrate into the fibrin clot and necrotic abscesses [12]. Empirical antibiotics of choice include penicillin/β-lactamase inhibitor, penicillin plus metronidazole, or a carbapenem. The use of anticoagulation to prevent propagation of thrombi and/or further embolization is controversial but is described in our case.

## Conclusions

Lemierre’s syndrome is an increasingly recognized and potentially fatal condition in otherwise healthy young individuals involving infective thrombophlebitis post tonsillar infection with associated embolic complications. A high clinical index of suspicion should prompt diagnostic investigations and early institution of treatment to reduce morbidity and mortality rates.
